# Toxicity of DON on GPx1-Overexpressed or Knockdown Porcine Splenic Lymphocytes In Vitro and Protective Effects of Sodium Selenite

**DOI:** 10.1155/2019/5769752

**Published:** 2019-02-28

**Authors:** Zhihua Ren, Changhao Chen, Yu Fan, Chaoxi Chen, Hongyi He, Xuemei Wang, Zhuo Zhang, Zhicai Zuo, Guangneng Peng, Yanchun Hu, Zhiwen Xu, Siyi Tao, Xinru Mao, Junliang Deng

**Affiliations:** ^1^College of Veterinary Medicine, Sichuan Province Key Laboratory of Animal Disease and Human Health, Key Laboratory of Environmental Hazard and Human Health of Sichuan Province, Sichuan Agricultural University, Chengdu 611130, China; ^2^College of Life Since and Technology, Southwest Minzu University, Chengdu, Sichuan 610041, China

## Abstract

Deoxynivalenol (DON) is a common contaminant of grain worldwide and is often detected in the human diet and animal feed. Selenium is an essential trace element in animals. It has many biological functions. The role of selenium in the body is mainly orchestrated by selenoprotein. Glutathione peroxidase (GPx) also exists widely in the body and has attracted much attention due to its high antioxidant capacity. In order to explore the effect of the GPx1 gene on toxicity of DON, in this study, we overexpressed or knockdown GPx1 in porcine splenic lymphocytes, then added different concentrations of DON (0.1025, 0.205, 0.41, and 0.82 *μ*g/mL) and sodium selenite (2 *μ*mol/L) to the culture system. Using various techniques, we detected antioxidant function, free radical content, cell apoptosis, and methylation-related gene expression to explore the effect of GPx1 expression on DON-induced cell damage. We also explored whether selenium can antagonize the toxicity of DON in these two cell models and revealed the protective effect of sodium selenite on DON-induced cell damage in GPx1-overexpressing or knockdown splenic lymphocytes. Finally, our findings revealed the following: (1) GPx1 can regulate the antioxidant capacity, apoptosis rate, and expression of DNA methylation-related genes in pig splenic lymphocytes. (2) Na_2_SeO_3_ (2 *μ*mol/L) can regulate the antioxidant capacity, apoptosis rate, and expression of DNA methylation-related genes in pig splenic lymphocytes, and this effect is more significant in GPx1-overexpressing cells than in GPx1-knockdown cells. (3) DON can cause oxidative damage, apoptosis, and methylation injury in GPx1-overexpressing or knockdown pig splenic lymphocytes in a concentration-dependent manner. (4) Na_2_SeO_3_ (2 *μ*mol/L) can antagonize the toxic effect of DON on GPx1-overexpressing or knockdown pig splenic lymphocytes. Our findings may have important implications for food/feed safety, human health, and environmental protection.

## 1. Introduction

Mycotoxin is a secondary metabolite of mold. It can exist in grain during various stages of harvesting and food processing and is generally stable and therefore not easily destroyed or removed. It can lead to a variety of toxic symptoms in humans and animals contaminated with mycotoxin [1]. Common fungi that contaminate cereals and other foods include *Penicillium*, *Aspergillus*, and *Fusarium* [2]. *Fusarium* toxins are the largest group of mycotoxins that affect the global food industry, the feed industry, and the aquaculture industry [3]. DON is a *Trichoderma* toxin, mainly produced by *Fusarium graminearum*. It is a common cause of food contamination. Different species of animals have different levels of tolerance to DON, with pigs being among the most sensitive [4]. DON can not only reduce the utilization rate of animal feed but also reduce the growth and reproductive performance of animals, as well as destroying their immune system.

Deoxynivalenol is also known as vomit toxin because it interacts with the 5-serotonin and dopamine receptors located in the vomit center of the brain stem. Consumption of food contaminated by DON therefore causes acute poisoning symptoms such as vomiting, diarrhea, and anorexia [5]. Studies have shown that DON can disrupt the transduction of cell information, cell differentiation, cell growth, and synthesis of macromolecular substances [4]. DON can act on the immune system of the body, resulting in immunosuppression and lowering the body's immunity.

DON is a serious source of global pollution. The detection rate of DON in fresh harvested wheat in Brazil was 99%, and the exceeding standard rate was 10% [6]. The detection rate of DON in plant-derived foods in Czech and the United States is 78% [7].

During the process of peroxidation, lipids produce many free radicals and non-free radical products, resulting in cell dysfunction. Among them, the lipid free radicals of medium reactivity are easily diffused into the nucleus, where they react with a base and directly affect DNA and RNA, causing gene mutations and leading to cell carcinogenesis. A study found that the oxidative damage caused by *Fusarium* toxins is likely to be due to destruction of the body's antioxidant system and acceleration of the production of free radicals [8], which has been confirmed in Caco-2 and HepG2 cells [9]. Ren and colleagues [10] used 0.15 mg/mL DON to poison Kunming mice several times and found that the concentration of superoxide dismutase (SOD) and glutathione (GSH) in the brains of affected mice was significantly lower than that in the normal group. DON can reduce the total antioxidant capacity of cells and the ability of cells to inhibit hydroxyl radicals. Thus, whether *in vivo* or *in vitro*, the toxicity of DON is closely related to the induction oxidative stress.

Previous studies have shown that DON can interfere with and damage ribosomes [11], inhibit the synthesis of protein and nucleic acids [12, 13], and promote apoptosis [14, 15]. Oxidative stress is an important mechanism of DON-mediated cytotoxicity and apoptosis [16]. The main mechanism by which DON induces oxidative stress is via the accumulation of a large amount of reactive oxygen species (ROS) in the cell, which disrupts the balance between oxidation and antioxidant activity in the cells. ROS accumulation causes lipid peroxidation on the lipid membrane and damage to the phospholipids and lipoprotein on the membrane, in turn damaging the DNA by a transmission chain reaction [17]. Oxidative stress in cells can result in oxidative damage and promotes cell apoptosis [15].

A high dose of DON can cause immune suppression, resulting from the DON-induced apoptosis of immune cells and inhibition of protein translation. A high dose of DON can damage the immune system and induce the apoptosis of leukocyte, macrophages, T cells, and B cells. In pigs, DON can not only compromise animal immunity but also lead to the repeated outbreak of diseases [18].

The spleen, as the target organ of DON, plays an important role in DON-mediated effects. DON can change the morphological structure of the spleen and affect the function of spleen. When the spleen structure changes and is damaged, immune function is also affected. When DON acts on spleen lymphocytes, it can cause oxidative damage to cells, affect the expression of immunoglobulins, and change the antioxidant index. Ren and colleagues [10] administered mice with DON at 2.5 mg/kg body weight on days 3, 5, 8, and 12. In a study of the changes in the antioxidant index of spleen lymphocytes induced by DON, Ren and colleagues [18] used DON at different doses (0.006, 0.3, 1.5, and 7.5 *μ*g/mL) to direct the pig spleen lymphocytes and found that the SOD, catalase (CAT), glutathione peroxidase (GPx), and GSH content decreased significantly compared with the control group, whereas the malondialdehyde (MDA) content increased significantly.

Selenium has many biological functions in organisms, the most important of which is its antioxidant effect. The biological function of selenium is mainly mediated by selenoprotein, which exists in the form of selenocysteine. GPx is an important peroxide decomposition enzyme that is widely distributed in the body. Selenium is a component of GPx and can enhance its vitality. GPx has strong antioxidant capacity. GPx can catalyze reduced GSH into oxidized glutathione (GSSG), reduce toxic peroxide (ROOH) into harmless hydroxyl compounds, and decompose hydrogen peroxide (H_2_O_2_), so as to protect the structure and function of the cell membrane from peroxide interference and damage. Studies have shown that there are four types of GPx, one of which is the cell type glutathione peroxidase widely found in various cells, also known as GPx1.

Studies have shown that selenium has the effect of antagonizing DON toxicity. Placha and coworkers [19] added 3 mg/kg DON toxin to a broiler diet and added excessive organic selenium (1 mg/kg) to the selenium test group to observe the antagonistic effect of selenium on DON. The results showed that a subtoxic dose of DON did not cause any clinical symptoms, but the activity of SOD was reduced, the MDA content was increased, and GPx activity was increased. The selenium test group significantly decreased the toxicity of DON, increased the activity of SOD, reduced the content of MDA, and significantly increased the activity of GPx in the tissues, and the organic selenium regulated the abnormal activity of GPx in the chicken duodenum caused by DON.

To sum up, we intend to overexpress or knock down the GPx1 gene of porcine spleen lymphocytes cultured *in vitro* and then use different concentrations of DON to expose it. Then, we observe whether there is any change in the antagonism of selenium to DON when GPx1 is too much or too little.

## 2. Materials and Methods

### 2.1. Cell Treatments and Grouping

The pig spleen was collected from 6-month-old Duchang commercial pigs from the Daxing pig slaughterhouse in Ya'an, Sichuan, China.

The pig spleen was aseptically extracted and after a few minutes was soaked in 75% alcohol. It was then washed three times in precooled PBS, and the connective tissue around the spleen was removed. After another two washes in precooled PBS, the remaining spleen was cut and transferred to a stainless steel screen (stainless steel screen (200) fixed on a petri dish containing 10 mL of PBS). Then, using the inner core of a disposable syringe, the spleen was gently squeezed into the PBS solution and the splenic lymphocyte suspension was adjusted with PBS solution to the desired concentration. Then, the cell suspension was slowly added into the glass centrifuge tube, which was prefitted with the splenic lymphocyte separation solution, and the cell suspension was slowly moved into the upper layer of the separated splenic lymphocytes at a 1 : 1 volume ratio. After centrifugation at 400 × *g* for 20 min, the supernatant was discarded and the middle layer of lymphocytes was transferred to another glass centrifuge tube. Five volumes of precooled PBS solution were added, the mixture was centrifuged at 250 × *g* for 10 min, and the cells were washed two more times in PBS then once in RPMI-1640 complete culture media (containing 10% fetal bovine serum). The cells were finally suspended in RPMI-1640 complete culture media, plated, trypan blue-stained, and counted. The cell concentration was 3.75 × 10^6^/mL, and the cell survival rate was over 95%. The cells could therefore be used for subsequent experiments.

According to the results of previous laboratory tests [20], the concentration of DON was determined to be 1/8 IC50, 1/4 IC50, 1/2 IC50, and IC50 (0.1025, 0.205, 0.41, and 0.82 *μ*g/mL), and Na_2_SeO_3_ concentration was the best concentration of 2 *μ*mol/L. The cells were grouped as shown in Table 1. DON was purchased from Sigma-Aldrich (CAS Number 51481-10-8, #D0156), and Na_2_SeO_3_ was purchased from Sigma-Aldrich (CAS Number 10102-18-8, #S9133).

### 2.2. Establishment of GPx1-Overexpressing Pig Splenic Lymphocytes *In Vitro*

#### 2.2.1. Construction and Verification of the Recombinant Plasmid pEGFP-N1-GPx1

**Figure 1:**
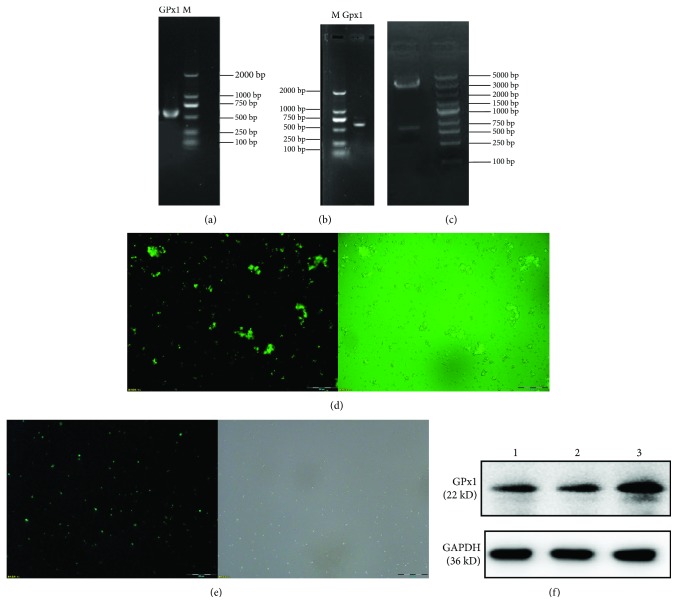
Establishment of GPx1 gene-overexpressing model of pig splenic lymphocytes. (a) shows the result of PCR amplification of the GPx1 gene of porcine splenic lymphocytes; it shows the PCR products, showing that the length of the single target gene was 621 bp. The PCR-amplified GPx1 gene was inserted into PEGFP-N1, a eukaryotic vector with kanamycin resistance, to obtain the recombinant plasmid pEGFP-N1-GPx1. Positive colonies were screened on kanamycin-containing plates. To avoid false positives, the selected colonies were screened by PCR, as shown in (b). The positive colonies were then verified by *XhoI* and *SacII* double-enzyme digestion to obtain bands of 630 bp and 4700 bp, as shown in (c). The X-tremeGENE HP DNA transfection reagent was used to transfect the purified plasmid into pig spleen lymphocytes over 48 h, and the efficiency of transfection was detected by fluorescence inverted microscopy, as shown in (d and e). (f) is the result of protein immunoblotting for GPx1. In (f), 1 is the untreated control group, 2 is the pEGFP-N1-transfected empty vector group, and 3 is the pEGFP-N1-GPx1-transfected overexpressed group.

*(1) Amplification of GPx1*. The electrophoretic results showed that the total RNA extracted from the splenic lymphocytes was reverse-transcribed into cDNA and used as a template for the PCR amplification of the GPx1 gene. Agarose gel electrophoresis (1%) of the PCR products showed that the length of the single target gene was 621 bp, as shown in Figure 1. The sequence of the GPx1 primers used for the amplification was as follows: F: CTCGAGATGTGCGCCGCTCAGCGTTCCGCTG and R: CCGCGGGGCACTGCTAGGCTCCTGGGACA.

*(2) Construction and Verification of Recombinant Plasmid pEGFP-N1-GPx1*. The PCR-amplified GPx1 gene was inserted into PEGFP-N1, a eukaryotic vector with kanamycin resistance, to obtain the recombinant plasmid pEGFP-N1-GPx1. Positive colonies were screened on kanamycin-containing plates. To avoid false positives, the selected colonies were screened by PCR, as shown in Figure 1. The positive colonies were then verified by *XhoI* and *SacII* double-enzyme digestion to obtain bands of 630 bp and 4700 bp, as shown in Figure 1.

*(3) Sequencing of the pEGFP-N1-GPx1 Recombinant Plasmid*. The pEGFP-N1 plasmid is 4.7 kb in size, and the target gene GPx1 is 621 bp in size. The sequencing results confirmed the correct construction of the recombinant plasmid pEGFP-N1-GPx1.

#### 2.2.2. Establishment of the Transfection Model Using Recombinant Plasmid pEGFP-N1-GPx1 in Pig Spleen Lymphocytes

The X-tremeGENE HP DNA Transfection Reagent was used to transfect the purified plasmid into pig spleen lymphocytes over 48 h, and the efficiency of transfection was detected by fluorescence inverted microscopy, as shown in Figure 1.

### 2.3. Establishment of GPx1 Knockdown Pig Spleen Lymphocytes *In Vitro*

#### 2.3.1. Design of GPx1-siRNA

Based on the published pig GPx1 mRNA (GenBank accession number NM-214201.0), siRNAs were designed using the BLOCK-iT™ siRNAs software (Thermo Fisher Scientific). The positive siRNA sequence 5′-GGGACUACACCCAGAUGAATT-3′ and the negative siRNA sequence 5′-UUCGUAUCUGGGUGUACCCTT-3′ were synthesized by Thermo Fisher Scientific. A FAM fluorescence tag was added to the siRNA as needed.

#### 2.3.2. Establishment of a Transfection Model for GPx-1 Knockdown Pig Splenic Lymphocytes

Transfection was carried out with 10 *μ*L of RFect^PM^ primary cell nucleotide transfection reagent and GPx1-siRNA, and the transfection efficiency was detected by flow cytometry after 24 h of transfection.

### 2.4. Determination of Antioxidant Capacity

After transfection, cells were cultured for 48 h and then centrifuged to collect the supernatant or precipitate. ELISA kits (ELISA kit is purchased from Nanjing Jiancheng Bioengineering Institute, China) were employed to determine the antioxidant-related indexes in the cell supernatant or precipitant, including glutathione peroxidase (GSH-Px), GSH, SOD, total antioxidant capacity (T-AOC), CAT, MDA, H_2_O_2_, and ROS (flow cytometry was used to detect the fluorescence intensity of ROS). The absorbance at 405, 450, and 532 nm was measured using a spectrophotometer. RT-PCR was used to determine the mRNA expression of GPx1 and SOD. Each experiment set three repetitions.

### 2.5. Determination of Apoptosis

After transfection, cells were cultured for 48 h and were then harvested by centrifugation and stained with Annexin V FITC/PI (Nanjing Jiancheng Bioengineering Institute). The cell apoptosis rate was measured by flow cytometry (Accuri C6, BD Biosciences), and the mRNA expression level of Bcl-2 and Bax was detected by RT-PCR. Each experiment set three repetitions.

### 2.6. Detection of Genes Related to Methylation

RT-PCR was used to detect the mRNA expression of methylation-related genes, including methyltransferase DNMT1, DNMT3a, and DNMT3b and the demethylation gene MBD2. Each experiment set three repetitions.

### 2.7. Statistical Analysis

Using SPSS 22.0 software, Student's *t* test was used to compare statistics. Results are presented as mean ± SD.

## 3. Results

### 3.1. Establishment of the Transfection Model Using Recombinant Plasmid pEGFP-N1-GPx1 in Porcine Spleen Lymphocytes

The transfection efficiency of recombinant plasmid pEGFP-N1-GPx1 in porcine spleen lymphocytes was 62.41%, and expression of the GPx1 gene was detected by real-time fluorescent quantitative PCR, the results of which are presented in Table 2. The expression of GPx1 mRNA in the overexpressing group was 3.876 times higher than that in the control group. The results of protein immunoblotting for GPx1 are shown in Figure 1. The results are expressed by mean value ± standard deviation, and the mean variance analysis was used to compare the differences among groups as shown in Table 3. The results showed that there was no significant difference between the control group and the empty vector group. The expression level of GPx1 protein in the overexpression group was 1.570 times higher than that in the control group, confirming that it could be used in subsequent experiments.

### 3.2. Establishment of a Transfection Model for GPx-1 Knockdown Porcine Splenic Lymphocytes

**Figure 2:**
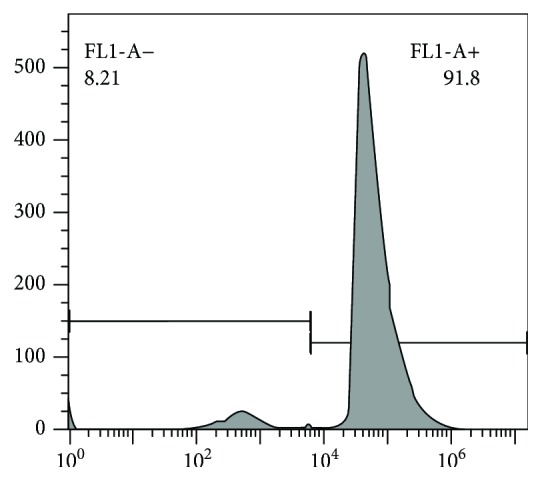
Transfection efficiency of GPx1-siRNA in porcine splenic lymphocytes. Transfection was carried out with 10 *μ*L of RFect^PM^ primary cell nucleotide transfection reagent and GPx1-siRNA, and the transfection efficiency was detected by flow cytometry after 24 h of transfection. The transfection efficiency of GPx1-siRNA in the spleen lymphocytes of pigs was 91.8%.

The transfection efficiency of GPx1-siRNA in the spleen lymphocytes of pigs was 91.8%, as shown in Figure 2. The expression of GPx1 mRNA was detected by real-time fluorescence quantitative PCR as shown in Table 4, and the expression of GPx1 mRNA in the silent group was 0.284 times that of the control group, confirming its suitability for use in subsequent experiments.

### 3.3. Related Index of the GPx1 Overexpression Group

#### 3.3.1. Effect of Na_2_SeO_3_ on DON-Induced Oxidative Toxicity of GPx1-Overexpressed Splenic Lymphocytes of Pigs

ELISA kit was used to detect the levels of GSH-Px, GSH, SOD, T-AOC, and CAT among the experimental groups shown in Table 1, to detect the MDA content and the presence of free radicals. The results are presented in Tables 5(a)–5(k). The GPx1-overexpressed type pig spleen lymphocyte cultured for 48 h, compared with the negative control group, the intracellular antioxidant index, the inhibition of hydroxyl free radical, and the mRNA expression of GPx1 and SOD were improved, and the levels of GSH-Px, T-AOC, and CAT increased significantly (*P* < 0.05). The contents of MDA, H_2_O_2_, and ROS decreased, and H_2_O_2_ decreased significantly (*P* < 0.05).

After adding Na_2_SeO_3_ alone, the antioxidant index and the expression of mRNA in the two genes were improved compared with the control (C) group. With the exception of SOD and T-AOC, the other indexes were significantly increased (*P* < 0.05 or *P* < 0.01). However, the contents of MDA, H_2_O_2_, and ROS showed a decreasing trend, and the content of free radicals decreased significantly (*P* < 0.01).

After adding DON alone, compared with the C group, the content of antioxidant index, the inhibition of hydroxyl free radical, and the expression of mRNA in the two genes were reduced, and the degree of decline increased with the increase in DON concentration, most of which were significantly decreased (*P* < 0.05 or *P* < 0.01). The contents of MDA, H_2_O_2_, and ROS all increased and showed an upward trend with the increase in the DON concentration, mostly showing a significant increase (*P* < 0.05 or *P* < 0.01).

After the combination of DON and Na_2_SeO_3_, the antioxidant capacity, the inhibition of hydroxyl radical ability, and the mRNA expression of two genes were improved, and most of them were significantly increased in the SD1 or SD2 group (*P* < 0.05). MDA, H_2_O_2_, and ROS all showed a downward trend, while MDA showed a significant decrease (*P* < 0.05).

#### 3.3.2. Effect of Na_2_SeO_3_ on DON-Induced Apoptosis of GPx1-Overexpressing Splenic Lymphocytes from Pigs

**Figure 3:**
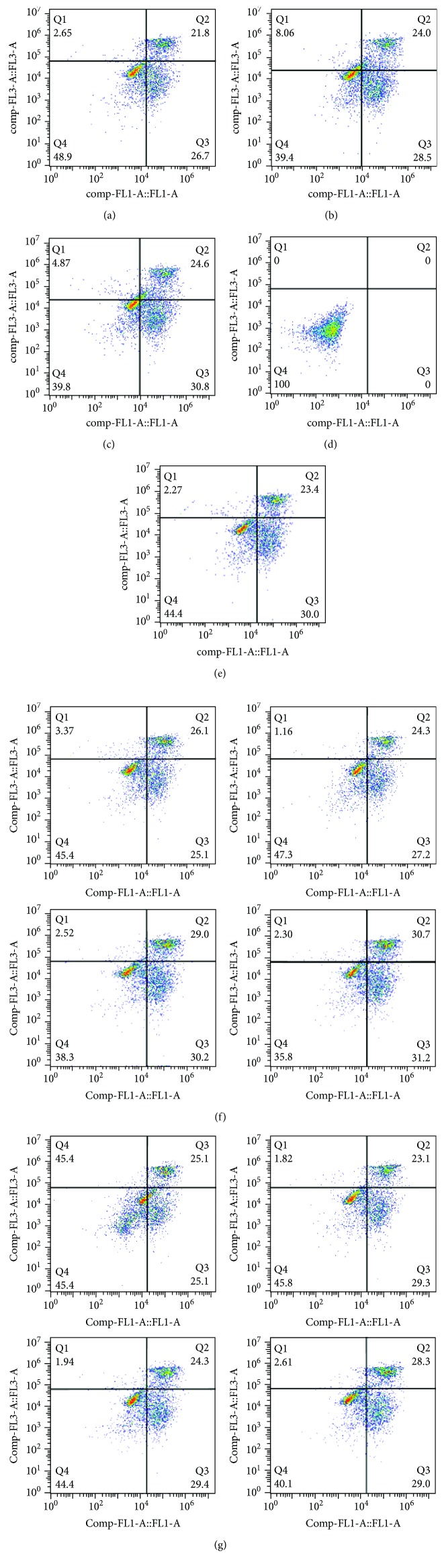
Apoptosis of GPx1 gene-overexpressing model in each group. (a) is the control group, (b) is the negative control, (c) is the empty vector group, (d) is the undyed group, (e) is the SE group, figure (f1-f4) are the D1-D4 groups, and figure (g1-g4) are the SD1-SD4 groups. In the images, the first and fourth quadrants show early apoptotic cells and late apoptotic cells, respectively, and the third quadrant shows normal cells.

Cells from the experimental group shown in Table 1 were cultured for 48 h and were then harvested and stained by Annexin V FITC/PI double staining. Flow cytometry was then used to detect the apoptosis rate, and the mRNA expression of Bcl-2 and Bax was detected by RT-PCR. Apoptotic cells are shown in Figure 3. In the images, the first and fourth quadrants show early apoptotic cells and late apoptotic cells, respectively, and the third quadrant shows normal cells. The survival rate of normal cells in group C was higher than that of the negative control and empty vector group. In groups D1–D4, the apoptosis rate increased with the increase in the DON concentration. After adding Na_2_SeO_3_, the apoptosis rate in the SD group was reduced. This suggested that Na_2_SeO_3_ can antagonize the DON-induced apoptosis of GPx1-overexpressing splenic lymphocytes in pigs.

The mRNA expression of apoptosis-related genes Bcl-2 and Bax is shown in Tables 6(a) and 6(b). The expression of Bcl-2 mRNA in the GPx1 overexpression group was higher than that of the negative control and empty vector groups. The mRNA expression of Bax was lower than that of the negative control and empty vector groups.

After the addition of 2 *μ*mol/L Na_2_SeO_3_, the expression of Bcl-2 and Bax was significantly increased or decreased (*P* < 0.05) compared with the control group.

After the addition of DON alone, the expression of Bcl-2 and Bax decreased or increased, respectively, compared with the control group, and in the other groups two genes were significantly reduced or elevated (*P* < 0.05), with the exception of Bcl-2 in the D1 group.

The addition of both DON and Na_2_SeO_3_ resulted in a further decrease in Bcl-2 expression and increase in Bax expression, and the expression in SD1 or SD2 was significant (*P* < 0.05).

#### 3.3.3. Effect of Na_2_SeO_3_ and DON on the Expression of Methylation-Related Genes in GPx1-Overexpressing Pig Splenic Lymphocytes

The mRNA expression of methyltransferase-related genes, including the methyltransferases DNMT1, DNMT3a, and DNMT3b and the demethylation enzyme MBD2, was detected by RT-PCR after 48 h of cultured of cells from the experimental groups detailed in Table 1. The results are shown in Tables 7(a)–7(d). The mRNA expression of the methyltransferases (DNMT1, DNMT3a, and DNMT3b) was higher in the control group than in the negative control or empty vector groups, whereas the expression of the demethylation enzyme (MBD2) was low.

After the addition of 2 *μ*mol/L Na_2_SeO_3_, the expression of the methyltransferase increased significantly (*P* < 0.05), whereas the expression of the demethylation enzyme decreased significantly (*P* < 0.05).

With the addition of DON, the expression of the methyltransferases was reduced compared with the control group, and the more the DON concentration increased, the lower the expression of methyltransferases, and DNMT1 decreased significantly (*P* < 0.01). Whereas the expression of the methylation enzymes increased in GPx1-overexpressing splenic lymphocytes following the addition of DON, and the more the DON concentration increased, the higher the expression of demethylation enzymes, with significant increases in the D3 and D4 groups (*P* < 0.05).

After the combined addition of DON and Na_2_SeO_3_, the expression of methyltransferase increased and the expression of demethylation enzyme decreased in comparison with the DON alone group.

### 3.4. Related Index of the GPx1 Knockdown Group

#### 3.4.1. Effect of Na_2_SeO_3_ on DON-Induced Oxidative Toxicity of the GPx1 Knockdown Splenic Lymphocytes of Pigs

ELISA kits were used to detect GSH-Px, GSH, SOD, T-AOC, CAT, and other antioxidant indexes and to detect the MDA content and free radical content in cells of the experimental groups detailed in Table 1. The results are shown in Tables 8(a)–8(k). After 48 h of culturing of the GPx1 knockdown pig splenic lymphocytes, the intracellular antioxidant index and the mRNA expression of GPx1 and SOD were reduced, most of them significantly (*P* < 0.05) compared with the negative control group. However, MDA, H_2_O_2_, and ROS levels increased significantly (*P* < 0.05 or *P* < 0.01).

After the addition of 2 *μ*mol/L Na_2_SeO_3_, the intracellular antioxidant index and the mRNA expression of GPx1 and SOD were increased, most significantly (*P* < 0.05) compared with the M group, while MDA, H_2_O_2_, and ROS expression was decreased, and the levels of ROS were significantly decreased (*P* < 0.01).

After the addition of DON alone, the intracellular antioxidant index, the inhibition of hydroxyl radicals and the mRNA expression of GPx1 and SOD decreased compared with the M group, and the higher the concentration of DON, the more significant the decrease (*P* < 0.05 or *P* < 0.01), and in the D1 group, GSH and GSH-Px were also decreased. By contrast, MDA, H_2_O_2_, and ROS levels increased, and the higher the DON concentration, the more significant the increase (*P* < 0.05 or *P* < 0.01). After the addition of both DON and Na_2_SeO_3_, the intracellular antioxidant index, the ability to inhibit hydroxyl radicals, and the mRNA expression of GPx1 and SOD were increased, and the SD1 and SD2 groups were significantly increased (*P* < 0.05) compared with the DON alone groups.

#### 3.4.2. Effect of Na_2_SeO_3_ on DON-Induced Apoptosis of GPx1 Knockdown Splenic Lymphocytes of Pigs

**Figure 4:**
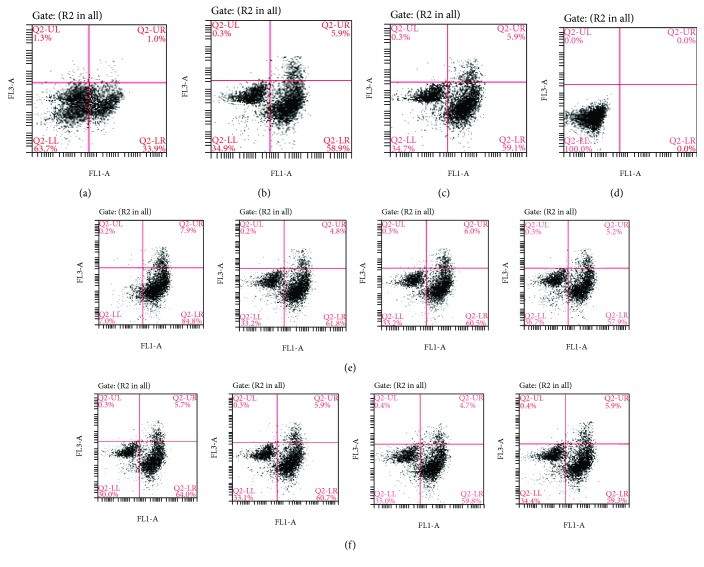
Apoptosis of the GPx1 gene knockdown model in each group. (a) is the P group (negative control group), (b) is the SE group, (c) is the M group (control group), (d) is the undyed group, (e) are D1-D4 groups, and (f) are SD1-SD4 groups. In the images, the first and fourth quadrants show early apoptotic cells and late apoptotic cells, respectively, and the third quadrant shows normal cells.

Cells from the experimental groups detailed in Table 1 were cultured for 48 h, then harvested and stained with Annexin V FITC/PI double staining. Flow cytometry was employed to detect the apoptosis rate, and the mRNA expression of Bcl-2 and Bax was detected by RT-PCR. Cell apoptosis is shown in Figure 4. In the images, the first and fourth quadrants show early apoptotic cells and late apoptotic cells, respectively, and the third quadrant shows normal cells. The results revealed that the survival rates of normal cells in group M were lower than those in group P. After the addition of DON, the apoptosis rate increased with the increase in the DON concentration. After the addition of Na_2_SeO_3_, the apoptosis rate in the SD groups was improved.

The mRNA expression of the apoptosis-related genes Bcl-2 and Bax in each of the groups is shown in Tables 9(a) and 9(b). The expression of Bcl-2 mRNA in group M was lower than that in group P, and the mRNA expression of Bax was higher than that in group P.

After the addition of 2 *μ*mol/L Na_2_SeO_3_, the expression of Bcl-2 and Bax increased or decreased, respectively, compared with the M group.

After the addition of DON, the expression of Bcl-2 and Bax decreased or increased, respectively, compared with the M group. By increasing the DON concentration, the decreased or increased expression of the two genes was more significant, indicating that DON could lead to the apoptosis of GPx1 knockdown pig splenic lymphocytes, and this effect was concentration-dependent.

After the addition of both DON and Na_2_SeO_3_, the decrease in Bcl-2 expression and the increase in Bax expression showed that Na_2_SeO_3_ could antagonize the apoptosis induced by DON in GPx1 knockdown spleen lymphocytes of pigs.

#### 3.4.3. Effect of Na_2_SeO_3_ on the Expression of Methylation-Related Genes Induces by DON in GPx1-Knockdown Pig Splenic Lymphocytes

Cells of the experimental groups detailed in Table 1 were cultured for 48 h, then the mRNA expression of methylation-related genes, including the methyltransferases DNMT1, DNMT3a, and DNMT3b and the demethylation enzyme MBD2, was detected by RT-PCR. The results are shown in Tables 10(a)–10(d). The expression of mRNA in group M (DNMT1, DNMT3a, and DNMT3b) is lower than that in the P group, and the expression of MBD2 was higher.

After the addition of 2 *μ*mol/L Na_2_SeO_3_, the expression of methyltransferases increased and the expression of the demethylation enzyme decreased.

Following the addition of DON alone, the expression of methyltransferases decreased compared with the M group, and this decrease was more significant as the DON concentration was increased, whereas the expression of the demethylation enzyme increased, and this increase was more significant as the DON concentration increased. In addition to the D1 and D2 groups, the other groups also showed a significant increase in methyltransferase expression (*P* < 0.05).

After the addition of both DON and Na_2_SeO_3_, the expression of methyltransferases increased and the expression of the demethylation enzyme decreased in comparison with the DON alone group.

## 4. Discussion

In this study, the GPx1 gene was overexpressed or knockdown in the splenic lymphocytes of pigs and the cells were then cultured with different concentrations of DON and/or 2 *μ*mol/L Na_2_SeO_3_. Then, the antioxidative function, the free radical content, the apoptosis rate, and the expression of methylation-related genes were determined. These experiments explored the effects of the GPx1 gene on the cytotoxicity induced by DON at the molecular level and revealed the protective effects of Na_2_SeO_3_ on cell injury induced by DON in GPx1-overexpressed or knockdown porcine splenic lymphocytes.

Selenium, an essential trace element, has many biological functions, such as improving the body's antioxidant capacity, regulating the immunity of the body, affecting the body's reproductive function, antagonizing the toxicity of toxic substances, and promoting growth. Selenoprotein plays a key role in mediating these effects [21]. Overexpression of GPx1 can protect cells from the damage caused by H_2_O_2_ and fatty peroxide and can also prevent apoptosis induced by H_2_O_2_. GPx1 has the potential to protect many tissues from oxidative damage. In this study, the overexpression of GPx1 enhanced the antioxidant capacity of splenic lymphocytes and reduced the apoptosis rate of the cells, whereas the antioxidant capacity of the cells was weakened after GPx1 knockdown, and the apoptosis rate of the cells increased. These two cell models provided evidence for the protective effect of GPx1 on pig splenic lymphocytes. Different concentrations of Na_2_SeO_3_ could upregulate the expression and activity of GPx mRNA [22]. The experimental results showed that the expression of mRNA in GPx1 was higher than that of normal pig splenic lymphocytes, suggesting that selenium (added in the form of 2 *μ*mol/L Na_2_SeO_3_) had a significant effect on GPx1 in splenic lymphocytes. GPx as an important peroxidase in the body, and selenium, in the form of selenoprotein, affects its function.

DON has been reported to upregulate the immune system at low concentration [15], whereas at high concentration it has been shown to have an immunosuppressive effect. This difference may be caused by the concentration and duration of DON exposure and the difference in sensitivity between animals. In this study, four concentrations of DON were added to GPx1-overexpressing or knockdown pig splenic lymphocytes *in vitro*, and different extents of oxidative damage were observed. Under the two high concentrations of D3 and D4, a significant reduction in the antioxidant indices was predominantly observed (*P* < 0.05), whereas this reduction was predominantly not significant (*P* > 0.05) under the two lower concentrations of D1 and D2. This likely indicates that pigs are sensitive to DON at higher concentrations.

Regarding cell apoptosis, DON can activate apoptosis-related genes, promoting the expression of Bax and Bid and inducing murine thymocytes and human colon cancer cells (HT29) to induce apoptosis [19, 23]. It has previously been reported that the addition of different concentrations of DON to porcine hepatocytes induced cell apoptosis after 6 h, and the higher the concentration of DON, the higher the rate of apoptosis [24]. Similarly, in our study, four different concentrations of DON induced apoptosis of GPx1-overexpressing or knockdown pig splenic lymphocytes, and the higher the concentration, the higher the rate of apoptosis, along with decreased mRNA expression of Bcl-2 and increased mRNA expression of Bax. However, the apoptosis rate in GPx1-overexpressing cells in groups D1–D4 was lower than that of GPx1-knockdown cells in these groups. Furthermore, the expression of Bcl-2 and Bax mRNA showed that overexpression of GPx1 could effectively alleviate the apoptosis induced by DON.

Regarding DNA methylation, DNMT3a, DNMT3b, and DNMT1 synergistically maintain the stability of DNA methylation [25], whereas MBD2 mediates demethylation. It has been reported that the aberrant gene expression may be related to DNA demethylation [26]. Most of the changes in DNA methylation are due to chemicals in food and in the environment. For example, the heavy metal arsenic can induce DNA injury in the liver of mice, and the level of genomic methylation is reduced [27] in a dose- and time-dependent manner [28]. Similarly, exposure of rat liver cells to cadmium led to decreased expression of DNMT and a decrease in total methylation [29]. Aberrant DNA methylation is also associated with tumorigenesis, because tumors are often associated with DNA mutations that lead to a decrease in total methylation or an increase in the methylation level of the CpG island in the promoter region. In rat hepatocytes, protooncogenes were found to have a low methylation status, which promoted the expression of oncogenes and the amplification of cancer cells [29]. In the current study, knockdown of GPx1 resulted in decreased expression of the DNMT genes and increased expression of MBD2, whereas GPx1 overexpression resulted in increased expression of DNMT and decreased expression of MBD2. This indicated that GPx1 can regulate the expression of methylation-related genes. Following exposure to 2 *μ*mol/L Na_2_SeO_3_, DNMT expression increased and MBD2 expression decreased, indicating that selenium could also regulate methylation-related genes. DON was shown to downregulate DNMT expression and upregulate MBD2 expression, and the higher the concentration of DON, the greater the effect. This may also implicate DON in the process of tumorigenesis. Selenium was shown to antagonize the effect of DON on DNA methylation.

In conclusion, overexpression of the GPx1 gene can reduce oxidative damage in porcine splenic lymphocytes, reduce the rate of apoptosis, and reduce the degree of DNA methylation. Following knockdown of the GPx1 gene, the opposite trends were observed including increased oxidative damage, increased apoptosis, and increased DNA methylation damage. However, independent of overexpression or knockdown, exposure of splenic lymphocytes to DON caused concentration-dependent oxidative stress, apoptosis, and DNA methylation. Treatment with 2 *μ*mol/L Na_2_SeO_3_ enhanced mRNA expression of the GPx1 gene and antagonized the toxicity of DON, thereby improving the intracellular environment.

Our findings revealed the following: (1) GPx1 can regulate the antioxidant capacity, apoptosis rate, and expression of DNA methylation-related genes in pig splenic lymphocytes. (2) Na_2_SeO_3_ (2 *μ*mol/L) can regulate the antioxidant capacity, apoptosis rate, and expression of DNA methylation-related genes in pig splenic lymphocytes, and this effect is more significant in GPx1-overexpressing cells than in GPx1-knockdown cells. (3) DON can cause oxidative damage, apoptosis, and methylation injury in GPx1-overexpressing or knockdown pig splenic lymphocytes in a concentration-dependent manner. (4) Na_2_SeO_3_ (2 *μ*mol/L) can antagonize the toxic effect of DON on GPx1-overexpressing or knockdown pig splenic lymphocytes.

## Figures and Tables

**Table 1:** Experimental groups.

Models	GPx1 overexpression	GPx1 knockdown
Groups		
C/M	GPx1 overexpression cells	GPx1 knockdown cells
P	Negative control	Negative control
K	Empty carrier	
SE	*n*_Na2SeO3_ = 2 *μ*mol/L	*n*_Na2SeO3_ = 2 *μ*mol/L
D1	*n*_DON_ = 0.1025 *μ*g/mL	*n*_DON_ = 0.1025 *μ*g/mL
D2	*n*_DON_ = 0.205 *μ*g/mL	*n*_DON_ = 0.205 *μ*g/mL
D3	*n*_DON_ = 0.41 *μ*g/mL	*n*_DON_ = 0.41 *μ*g/mL
D4	*n*_DON_ = 0.82 *μ*g/mL	*n*_DON_ = 0.82 *μ*g/mL
SD1	SE + D1	SE + D1
SD2	SE + D2	SE + D2
SD3	SE + D3	SE + D3
SD4	SE + D4	SE + D4

**Table 2:** Expression of GPx1 mRNA after transfection of recombinant plasmid pEGFP-N1-GPx1 into porcine splenic lymphocytes.

Groups	Control	Empty vector	Overexpress
Gene expression	1.000 ± 0.135^a^	1.054 ± 0.128^a^	3.876 ± 0.158^b^

Data are presented as mean ± standard deviation. Different lowercase letters indicate a significant difference (*P* < 0.05); the same lowercase letters indicate no significant difference between the groups (*P* > 0.05).

**Table 3:** Expression of GPx1 protein after transfection of recombinant plasmid pEGFP-N1-GPx1 into porcine splenic lymphocytes.

Groups	Control	Empty vector	Overexpress
Protein expression	0.530 ± 0.002^a^	0.528 ± 0.002^a^	0.832 ± 0.006^b^

Data are presented as mean ± standard deviation. Different lowercase letters indicate a significant difference (*P* < 0.05); the same lowercase letters indicate no significant difference between the groups (*P* > 0.05).

**Table 4:** The relative expression of GPx1-mRNA.

Groups	Knockdown	Negative control	Control
Gene expression	0.284 ± 0.032	0.994 ± 0.048	1 ± 0.017

**Table:** (a) Activities of GSH-Px in GPx1 overexpressed splenic lymphocytes of pigs

GSH-Px				
Time	Groups	Activities (U/mg·prot)		*P*
48 h	C-P	149.755 ± 3.947	136.628 ± 2.093	0.044
	P-K	136.628 ± 2.093	133.877 ± 4.176	0.500
	C-K	149.755 ± 3.947	133.877 ± 4.176	0.060
	C-SE	149.755 ± 3.947	172.064 ± 5.876	0.050
	C-D1	149.755 ± 3.947	130.976 ± 11.275	0.081
	C-D2	149.755 ± 3.947	91.903 ± 4.625	0.007
	C-D3	149.755 ± 3.947	76.925 ± 8.191	0.009
	C-D4	149.755 ± 3.947	58.002 ± 5.562	0.002
	D1-SD1	130.976 ± 11.275	102.959 ± 3.057	0.068
	D2-SD2	91.903 ± 4.625	117.725 ± 6.960	0.009
	D3-SD3	76.925 ± 8.191	89.450 ± 8.310	0.257
	D4-SD4	58.002 ± 5.562	86.610 ± 7.977	0.056

**Table:** (b) Contents of GSH in GPx1-overexpressed splenic lymphocytes of pigs

GSH				
Time	Groups	Contents (*μ*mol/g·prot)		*P*
48 h	C-P	1059.729 ± 163.521	937.428 ± 50.554	0.295
	P-K	937.428 ± 50.554	1035.283 ± 256.003	0.583
	C-K	1059.729 ± 163.521	1035.283 ± 256.003	0.767
	C-SE	1059.729 ± 163.521	1474.090 ± 28.556	0.048
	C-D1	1059.729 ± 163.521	548.910 ± 291.210	0.170
	C-D2	1059.729 ± 163.521	278.067 ± 2.741	0.014
	C-D3	1059.729 ± 163.521	370.592 ± 116.622	0.002
	C-D4	1059.729 ± 163.521	259.504 ± 19.445	0.011
	D1-SD1	548.910 ± 291.210	633.252 ± 41.600	0.703
	D2-SD2	278.067 ± 2.741	624.508 ± 42.319	0.004
	D3-SD3	370.592 ± 116.622	461.139 ± 9.442	0.339
	D4-SD4	259.504 ± 19.445	310.588 ± 10.997	0.039

**Table:** (c) Activities of SOD in GPx1-overexpressed splenic lymphocytes of pigs

SOD				
Time	Groups	Activities (U/mg·prot)		*P*
48 h	C-P	72.263 ± 1.755	71.474 ± 2.080	0.165
	P-K	71.474 ± 2.080	71.278 ± 0.956	0.848
	C-K	72.263 ± 1.755	71.278 ± 0.956	0.387
	C-SE	72.263 ± 1.755	76.221 ± 1.164	0.130
	C-D1	72.263 ± 1.755	69.062 ± 0.555	0.057
	C-D2	72.263 ± 1.755	67.015 ± 1.323	0.094
	C-D3	72.263 ± 1.755	58.557 ± 1.277	0.008
	C-D4	72.263 ± 1.755	39.224 ± 8.728	0.024
	D1-SD1	69.062 ± 0.555	77.838 ± 1.006	0.010
	D2-SD2	67.015 ± 1.323	72.383 ± 2.741	0.078
	D3-SD3	58.557 ± 1.277	62.831 ± 1.737	0.133
	D4-SD4	39.224 ± 8.728	64.137 ± 8.864	0.003

**Table:** (d) Activities of T-AOC in GPx1-overexpressed splenic lymphocytes of pigs

T-AOC				
Time	Groups	Activities (U/mg·prot)		*P*
48 h	C-P	0.650 ± 0.012	0.500 ± 0.006	0.004
	P-K	0.500 ± 0.006	0.500 ± 0.001	0.959
	C-K	0.650 ± 0.012	0.500 ± 0.001	0.002
	C-SE	0.650 ± 0.012	0.700 ± 0.022	0.112
	C-D1	0.650 ± 0.012	0.488 ± 0.012	0.007
	C-D2	0.650 ± 0.012	0.480 ± 0.008	0.002
	C-D3	0.650 ± 0.012	0.473 ± 0.025	0.012
	C-D4	0.650 ± 0.012	0.461 ± 0.008	0.003
	D1-SD1	0.488 ± 0.012	0.508 ± 0.016	0.274
	D2-SD2	0.480 ± 0.008	0.485 ± 0.022	0.716
	D3-SD3	0.473 ± 0.025	0.480 ± 0.004	0.708
	D4-SD4	0.461 ± 0.008	0.477 ± 0.003	0.107

**Table:** (e) Activities of CAT in GPx1-overexpressed splenic lymphocytes of pigs

CAT				
Time	Groups	Activities (U/mg·prot)		*P*
48 h	C-P	31.499 ± 1.047	29.691 ± 1.645	0.035
	P-K	29.691 ± 1.645	29.291 ± 1.402	0.288
	C-K	31.499 ± 1.047	29.291 ± 1.402	0.013
	C-SE	31.499 ± 1.047	32.153 ± 1.078	0.005
	C-D1	31.499 ± 1.047	14.021 ± 0.514	0.001
	C-D2	31.499 ± 1.047	10.298 ± 0.394	0.001
	C-D3	31.499 ± 1.047	8.053 ± 0.982	0.002
	C-D4	31.499 ± 1.047	5.224 ± 0.442	0.001
	D1-SD1	14.021 ± 0.514	15.362 ± 1.739	0.404
	D2-SD2	10.298 ± 0.394	13.183 ± 0.888	0.058
	D3-SD3	8.053 ± 0.982	9.977 ± 1.570	0.121
	D4-SD4	5.224 ± 0.442	8.985 ± 0.921	0.037

**Table:** (f) Contents of MDA in GPx1-overexpressing splenic lymphocytes of pigs

MDA				
Time	Groups	Contents (nmol/mg·prot)		*P*
48 h	C-P	8.570 ± 0.520	9.985 ± 0.097	0.040
	P-K	9.985 ± 0.097	10.000 ± 0.097	0.905
	C-K	8.570 ± 0.520	10.000 ± 0.097	0.038
	C-SE	8.570 ± 0.520	8.184 ± 0.298	0.253
	C-D1	8.570 ± 0.520	9.433 ± 0.393	0.008
	C-D2	8.570 ± 0.520	10.632 ± 0.260	0.010
	C-D3	8.570 ± 0.520	11.402 ± 0.476	0.027
	C-D4	8.570 ± 0.520	12.334 ± 0.462	0.006
	D1-SD1	9.433 ± 0.393	10.621 ± 0.324	0.006
	D2-SD2	10.632 ± 0.260	10.160 ± 0.366	0.016
	D3-SD3	11.402 ± 0.476	11.079 ± 0.240	0.481
	D4-SD4	12.334 ± 0.462	11.506 ± 0.446	0.045

**Table:** (g) Levels of Inhibition of hydroxyl radical in GPx1-overexpressed splenic lymphocytes of pigs

Inhibition of hydroxyl radical				
Time	Groups			*P*
48 h	C-P	180.220 ± 14.977	186.215 ± 14.746	0.001
	P-K	186.215 ± 14.746	203.751 ± 13.955	0.001
	C-K	180.220 ± 14.977	203.751 ± 13.955	0.001
	C-SE	180.220 ± 14.977	175.669 ± 13.755	0.024
	C-D1	180.220 ± 14.977	194.664 ± 22.824	0.106
	C-D2	180.220 ± 14.977	183.099 ± 14.274	0.001
	C-D3	180.220 ± 14.977	186.210 ± 14.970	0.001
	C-D4	180.220 ± 14.977	165.723 ± 14.095	0.039
	D1-SD1	194.664 ± 22.824	203.556 ± 13.937	0.233
	D2-SD2	183.099 ± 14.274	194.395 ± 14.171	0.004
	D3-SD3	186.210 ± 14.970	188.607 ± 13.768	0.225
	D4-SD4	165.723 ± 14.095	180.603 ± 13.500	0.006

**Table:** (h) Contents of H_2_O_2_ in GPx1-overexpressed splenic lymphocytes of pigs

H_2_O_2_				
Time	Groups	Contents (mmol/g·prot)		*P*
48 h	C-P	19.162 ± 0.617	20.580 ± 0.431	0.142
	P-K	20.580 ± 0.431	21.067 ± 0.418	0.001
	C-K	19.162 ± 0.617	21.067 ± 0.418	0.085
	C-SE	19.162 ± 0.617	18.872 ± 0.760	0.742
	C-D1	19.162 ± 0.617	24.379 ± 0.584	0.001
	C-D2	19.162 ± 0.617	26.283 ± 0.357	0.001
	C-D3	19.162 ± 0.617	26.412 ± 1.120	0.002
	C-D4	19.162 ± 0.617	28.531 ± 0.293	0.001
	D1-SD1	24.379 ± 0.584	21.565 ± 1.010	0.092
	D2-SD2	26.283 ± 0.357	23.850 ± 0.525	0.008
	D3-SD3	26.412 ± 1.120	22.938 ± 0.302	0.032
	D4-SD4	28.531 ± 0.293	23.285 ± 0.387	0.002

**Table:** (i) Levels of ROS in GPx1-overexpressed splenic lymphocytes of pigs

ROS				
Time	Groups			*P*
48 h	C-P	2185.667 ± 27.227	2304.333 ± 73.214	0.064
	P-K	2304.333 ± 73.214	2204.333 ± 49.642	0.270
	C-K	2185.667 ± 27.227	2204.333 ± 49.642	0.644
	C-SE	2185.667 ± 27.227	2028.000 ± 38.510	0.003
	C-D1	2185.667 ± 27.227	2147.000 ± 51.507	0.005
	C-D2	2185.667 ± 27.227	2675.667 ± 101.535	0.009
	C-D3	2185.667 ± 27.227	2824.667 ± 55.103	0.005
	C-D4	2185.667 ± 27.227	3246.333 ± 140.927	0.052
	D1-SD1	2147.000 ± 51.507	2358.667 ± 38.175	0.372
	D2-SD2	2675.667 ± 101.535	2469.667 ± 93.511	0.196
	D3-SD3	2824.667 ± 55.103	2503.667 ± 19.655	0.013
	D4-SD4	3246.333 ± 140.927	2714.000 ± 25.515	0.159

**Table:** (j) Expression of GPx1 in GPx1-overexpressed splenic lymphocytes of pigs

GPx1 mRNA				
Time	Groups	Contents		*P*
48 h	C-P	1.000 ± 0.078	0.914 ± 0.095	0.378
	P-K	0.914 ± 0.095	0.913 ± 0.028	0.983
	C-K	1.000 ± 0.078	0.913 ± 0.028	0.100
	C-SE	1.000 ± 0.078	5.420 ± 0.317	0.003
	C-D1	1.000 ± 0.078	1.038 ± 0.110	0.753
	C-D2	1.000 ± 0.078	0.913 ± 0.390	0.695
	C-D3	1.000 ± 0.078	0.704 ± 0.158	0.069
	C-D4	1.000 ± 0.078	0.515 ± 0.131	0.040
	D1-SD1	1.038 ± 0.110	3.423 ± 0.043	≤0.001
	D2-SD2	0.913 ± 0.390	2.862 ± 0.449	0.004
	D3-SD3	0.704 ± 0.158	2.351 ± 0.129	0.004
	D4-SD4	0.515 ± 0.131	2.127 ± 0.402	0.030

**Table:** (k) Expression of SOD in GPx1-overexpressed splenic lymphocytes of pigs

SOD mRNA				
Time	Groups	Contents		*P*
48 h	C-P	1.000 ± 0.118	0.906 ± 0.099	0.518
	P-K	0.906 ± 0.099	0.986 ± 0.057	0.343
	C-K	1.000 ± 0.118	0.986 ± 0.057	0.888
	C-SE	1.000 ± 0.118	4.757 ± 0.753	0.015
	C-D1	1.000 ± 0.118	0.450 ± 0.057	0.010
	C-D2	1.000 ± 0.118	0.293 ± 0.024	0.007
	C-D3	1.000 ± 0.118	0.262 ± 0.021	0.010
	C-D4	1.000 ± 0.118	0.171 ± 0.035	0.011
	D1-SD1	0.450 ± 0.057	0.675 ± 0.024	0.039
	D2-SD2	0.293 ± 0.024	0.501 ± 0.056	0.009
	D3-SD3	0.262 ± 0.021	0.454 ± 0.037	0.010
	D4-SD4	0.171 ± 0.035	0.232 ± 0.032	0.041

**Table:** (a) Expression of Bcl-2 in GPx1-overexpressed splenic lymphocytes of pigs

Bcl-2 mRNA				
Time	Groups	Contents		*P*
48 h	C-P	1.000 ± 0.018	0.957 ± 0.115	0.617
	P-K	0.957 ± 0.115	0.913 ± 0.101	0.396
	C-K	1.000 ± 0.018	0.913 ± 0.101	0.278
	C-SE	1.000 ± 0.018	1.190 ± 0.462	0.550
	C-D1	1.000 ± 0.018	0.953 ± 0.043	0.116
	C-D2	1.000 ± 0.018	0.699 ± 0.016	0.003
	C-D3	1.000 ± 0.018	0.574 ± 0.081	0.013
	C-D4	1.000 ± 0.018	0.670 ± 0.045	0.010
	D1-SD1	0.953 ± 0.043	0.947 ± 0.030	0.885
	D2-SD2	0.699 ± 0.016	0.811 ± 0.055	0.090
	D3-SD3	0.574 ± 0.081	0.664 ± 0.117	0.313
	D4-SD4	0.670 ± 0.045	0.827 ± 0.074	0.034

**Table:** (b) Expression of Bax in GPx1-overexpressed splenic lymphocytes of pigs

Bax mRNA				
Time	Groups	Contents		*P*
48 h	C-P	1.000 ± 0.049	1.311 ± 0.063	0.032
	P-K	1.311 ± 0.063	1.401 ± 0.126	0.336
	C-K	1.000 ± 0.049	1.401 ± 0.126	0.057
	C-SE	1.000 ± 0.049	1.437 ± 0.116	0.015
	C-D1	1.000 ± 0.049	1.716 ± 0.005	0.002
	C-D2	1.000 ± 0.049	1.781 ± 0.249	0.045
	C-D3	1.000 ± 0.049	2.317 ± 0.421	0.039
	C-D4	1.000 ± 0.049	4.410 ± 0.903	0.023
	D1-SD1	1.716 ± 0.005	1.214 ± 0.077	0.008
	D2-SD2	1.781 ± 0.249	1.379 ± 0.098	0.109
	D3-SD3	2.317 ± 0.421	2.163 ± 0.245	0.362
	D4-SD4	4.410 ± 0.903	3.941 ± 0.660	0.248

**Table:** (a) Expression of DNMT1 in GPx1-overexpressed splenic lymphocytes of pigs

DNMT1 mRNA				
Time	Groups	Contents		*P*
48 h	C-P	1.000 ± 0.030	1.099 ± 0.091	0.263
	P-K	1.099 ± 0.091	0.988 ± 0.024	0.115
	C-K	1.000 ± 0.030	0.988 ± 0.024	0.665
	C-SE	1.000 ± 0.030	5.076 ± 0.635	0.009
	C-D1	1.000 ± 0.030	0.695 ± 0.038	≤0.001
	C-D2	1.000 ± 0.030	0.328 ± 0.028	0.003
	C-D3	1.000 ± 0.030	0.294 ± 0.010	≤0.001
	C-D4	1.000 ± 0.030	0.263 ± 0.053	0.003
	D1-SD1	0.695 ± 0.038	0.702 ± 0.004	0.791
	D2-SD2	0.328 ± 0.028	0.802 ± 0.210	0.050
	D3-SD3	0.294 ± 0.010	0.515 ± 0.019	0.004
	D4-SD4	0.263 ± 0.053	0.377 ± 0.029	0.042

**Table:** (b) Expression of DNMT3a in GPx1-overexpressed splenic lymphocytes of pigs

DNMT3a mRNA				
Time	Groups	Contents		*P*
48 h	C-P	1.000 ± 0.153	0.930 ± 0.073	0.737
	P-K	0.930 ± 0.073	0.960 ± 0.068	0.069
	C-K	1.000 ± 0.153	0.960 ± 0.068	0.839
	C-SE	1.000 ± 0.153	7.267 ± 0.338	0.013
	C-D1	1.000 ± 0.153	4.386 ± 0.331	0.024
	C-D2	1.000 ± 0.153	2.804 ± 0.210	0.090
	C-D3	1.000 ± 0.153	1.833 ± 0.186	0.018
	C-D4	1.000 ± 0.153	1.633 ± 0.034	0.131
	D1-SD1	4.386 ± 0.331	0.617 ± 0.024	0.037
	D2-SD2	2.804 ± 0.210	0.504 ± 0.013	0.039
	D3-SD3	1.833 ± 0.186	0.465 ± 0.008	0.059
	D4-SD4	1.633 ± 0.034	0.441 ± 0.022	0.005

**Table:** (c) Expression of DNMT3b in GPx1-overexpressed splenic lymphocytes of pigs

DNMT3b mRNA				
Time	Groups	Contents		*P*
48 h	C-P	1.000 ± 0.043	0.928 ± 0.043	0.448
	P-K	0.928 ± 0.043	0.902 ± 0.010	0.609
	C-K	1.000 ± 0.043	0.902 ± 0.010	0.152
	C-SE	1.000 ± 0.043	29.478 ± 3.091	0.048
	C-D1	1.000 ± 0.043	0.842 ± 0.021	0.065
	C-D2	1.000 ± 0.043	0.670 ± 0.087	0.173
	C-D3	1.000 ± 0.043	0.648 ± 0.014	0.038
	C-D4	1.000 ± 0.043	0.571 ± 0.010	0.056
	D1-SD1	0.842 ± 0.021	1.011 ± 0.071	0.133
	D2-SD2	0.670 ± 0.087	1.014 ± 0.050	0.174
	D3-SD3	0.648 ± 0.014	0.883 ± 0.036	0.096
	D4-SD4	0.571 ± 0.010	0.611 ± 0.004	0.161

**Table:** (d) Expression of MBD2 in GPx1-overexpressed splenic lymphocytes of pigs

MBD2 mRNA				
Time	Groups	Contents		*P*
48 h	C-P	1.000 ± 0.037	1.017 ± 0.098	0.817
	P-K	1.017 ± 0.098	1.005 ± 0.018	0.824
	C-K	1.000 ± 0.037	1.005 ± 0.018	0.867
	C-SE	1.000 ± 0.037	0.828 ± 0.150	0.011
	C-D1	1.000 ± 0.037	1.025 ± 0.214	0.875
	C-D2	1.000 ± 0.037	1.003 ± 0.051	0.951
	C-D3	1.000 ± 0.037	1.204 ± 0.097	0.040
	C-D4	1.000 ± 0.037	1.550 ± 0.045	0.006
	D1-SD1	0.828 ± 0.150	0.959 ± 0.028	0.205
	D2-SD2	1.003 ± 0.051	1.109 ± 0.185	0.418
	D3-SD3	1.204 ± 0.097	1.202 ± 0.026	0.979
	D4-SD4	1.550 ± 0.045	1.310 ± 0.110	0.028

**Table:** (a) Activities of GSH-Px in GPx1 knockdown splenic lymphocytes of pigs

GSH-Px				
Time	Groups	Activities (U/mg·prot)		*P*
48 h	P-M	54.885 ± 6.179	29.987 ± 5.632	0.063
	M-SE	29.987 ± 5.632	32.489 ± 0.613	0.557
	M-D1	29.987 ± 5.632	20.798 ± 2.102	0.122
	M-D2	29.987 ± 5.632	20.416 ± 4.341	0.228
	M-D3	29.987 ± 5.632	17.916 ± 7.971	0.121
	M-D4	29.987 ± 5.632	14.786 ± 2.330	0.019
	D1-SD1	20.798 ± 2.102	21.846 ± 1.947	0.672
	D2-SD2	20.416 ± 4.341	20.262 ± 2.084	0.967
	D3-SD3	17.916 ± 7.971	18.255 ± 0.506	0.951
	D4-SD4	14.786 ± 2.330	15.153 ± 4.511	0.932

**Table:** (b) Contents of GSH in GPx1 knockdown splenic lymphocytes of pigs

GSH				
Time	Groups	Contents (*μ*mol/g·prot)		*P*
48 h	P-M	229.503 ± 11.620	228.077 ± 4.450	0.854
	M-SE	228.077 ± 4.450	237.312 ± 4.258	0.207
	M-D1	228.077 ± 4.450	231.685 ± 3.120	0.496
	M-D2	228.077 ± 4.450	212.852 ± 4.571	0.081
	M-D3	228.077 ± 4.450	185.798 ± 8.687	0.023
	M-D4	228.077 ± 4.450	165.431 ± 12.815	0.024
	D1-SD1	231.685 ± 3.120	238.462 ± 2.589	0.028
	D2-SD2	212.852 ± 4.571	221.689 ± 4.435	0.001
	D3-SD3	185.798 ± 8.687	197.444 ± 3.358	0.103
	D4-SD4	165.431 ± 12.815	158.854 ± 6.908	0.302

**Table:** (c) Activities of SOD in GPx1 knockdown splenic lymphocytes of pigs

SOD				
Time	Groups	Activities (U/mg·prot)		*P*
48 h	P-M	34.637 ± 0.925	24.637 ± 0.975	0.012
	M-SE	24.637 ± 0.975	24.863 ± 1.174	0.815
	M-D1	24.637 ± 0.975	17.423 ± 0.584	0.008
	M-D2	24.637 ± 0.975	14.677 ± 0.256	0.005
	M-D3	24.637 ± 0.975	14.169 ± 0.286	0.001
	M-D4	24.637 ± 0.975	12.762 ± 0.372	0.004
	D1-SD1	17.423 ± 0.584	20.011 ± 0.537	0.005
	D2-SD2	14.677 ± 0.256	16.672 ± 0.350	0.028
	D3-SD3	14.169 ± 0.286	14.427 ± 0.349	0.547
	D4-SD4	12.762 ± 0.372	12.892 ± 0.247	0.213

**Table:** (d) Activities of T-AOC in GPx1 knockdown splenic lymphocytes of pigs

T-AOC				
Time	Groups	Activities (U/mg·prot)		*P*
48 h	P-M	1.042 ± 0.002	0.852 ± 0.030	0.007
	M-SE	0.852 ± 0.030	0.937 ± 0.007	0.059
	M-D1	0.852 ± 0.030	0.686 ± 0.022	0.027
	M-D2	0.852 ± 0.030	0.576 ± 0.012	0.001
	M-D3	0.852 ± 0.030	0.454 ± 0.013	0.003
	M-D4	0.852 ± 0.030	0.320 ± 0.015	0.001
	D1-SD1	0.686 ± 0.022	0.824 ± 0.012	0.003
	D2-SD2	0.576 ± 0.012	0.676 ± 0.014	0.014
	D3-SD3	0.454 ± 0.013	0.572 ± 0.007	0.001
	D4-SD4	0.320 ± 0.015	0.429 ± 0.007	0.007

**Table:** (e) Activities of CAT in GPx1 knockdown splenic lymphocytes of pigs

CAT				
Time	Groups	Activities (U/mg·prot)		*P*
48 h	P-M	8.060 ± 0.165	5.631 ± 0.014	0.002
	M-SE	5.631 ± 0.014	5.675 ± 0.014	0.110
	M-D1	5.631 ± 0.014	4.401 ± 0.017	≤0.001
	M-D2	5.631 ± 0.014	3.645 ± 0.027	≤0.001
	M-D3	5.631 ± 0.014	3.304 ± 0.019	≤0.001
	M-D4	5.631 ± 0.014	2.552 ± 0.020	≤0.001
	D1-SD1	4.401 ± 0.017	4.628 ± 0.014	0.006
	D2-SD2	3.645 ± 0.027	4.287 ± 0.017	≤0.001
	D3-SD3	3.304 ± 0.019	3.797 ± 0.015	≤0.001
	D4-SD4	2.552 ± 0.020	2.695 ± 0.017	≤0.001

**Table:** (f) Contents of MDA in GPx1 knockdown splenic lymphocytes of pigs

MDA				
Time	Groups	Contents (nmol/mg·prot)		*P*
48 h	P-M	9.885 ± 0.430	11.298 ± 0.021	0.031
	M-SE	11.298 ± 0.021	11.084 ± 0.486	0.534
	M-D1	11.298 ± 0.021	13.102 ± 0.603	0.037
	M-D2	11.298 ± 0.021	14.539 ± 0.233	0.002
	M-D3	11.298 ± 0.021	15.901 ± 0.731	0.009
	M-D4	11.298 ± 0.021	17.207 ± 0.163	≤0.001
	D1-SD1	13.102 ± 0.603	12.053 ± 0.005	0.096
	D2-SD2	14.539 ± 0.233	13.649 ± 0.117	0.017
	D3-SD3	15.901 ± 0.731	15.049 ± 0.741	0.005
	D4-SD4	17.207 ± 0.163	16.688 ± 0.122	0.016

**Table:** (g) Levels of Inhibition of hydroxyl radical in GPx1 knockdown splenic lymphocytes of pigs

Inhibition of hydroxyl radical				
Time	Groups			*P*
48 h	P-M	199.732 ± 8.081	201.506 ± 3.681	0.731
	M-SE	201.506 ± 3.681	199.956 ± 9.070	0.827
	M-D1	201.506 ± 3.681	206.674 ± 9.643	0.423
	M-D2	201.506 ± 3.681	197.239 ± 20.584	0.776
	M-D3	201.506 ± 3.681	199.811 ± 6.386	0.423
	M-D4	201.506 ± 3.681	198.742 ± 12.815	0.778
	D1-SD1	206.674 ± 9.643	212.721 ± 19.732	0.423
	D2-SD2	197.239 ± 20.584	199.197 ± 15.383	0.769
	D3-SD3	199.811 ± 6.386	198.018 ± 7.108	0.733
	D4-SD4	198.742 ± 12.815	201.506 ± 3.681	0.778

**Table:** (h) Contents of H_2_O_2_ in GPx1 knockdown splenic lymphocytes of pigs

H_2_O_2_				
Time	Groups	Contents (mmol/g·prot)		*P*
48 h	P-M	96.215 ± 4.892	109.305 ± 2.377	0.013
	M-SE	109.305 ± 2.377	96.364 ± 4.274	0.007
	M-D1	109.305 ± 2.377	122.918 ± 4.504	0.008
	M-D2	109.305 ± 2.377	136.031 ± 1.673	0.001
	M-D3	109.305 ± 2.377	148.443 ± 5.391	0.002
	M-D4	109.305 ± 2.377	170.133 ± 3.329	≤0.001
	D1-SD1	122.918 ± 4.504	109.427 ± 3.406	0.005
	D2-SD2	136.031 ± 1.673	126.246 ± 3.673	0.014
	D3-SD3	148.443 ± 5.391	141.616 ± 4.469	0.029
	D4-SD4	170.133 ± 3.329	164.743 ± 5.516	0.064

**Table:** (i) Levels of ROS in GPx1 knockdown splenic lymphocytes of pigs

ROS				
Time	Groups			*P*
48 h	P-M	847.000 ± 10.869	1069.000 ± 22.847	0.006
	M-SE	1069.000 ± 22.847	893.330 ± 15.333	0.006
	M-D1	1069.000 ± 22.847	1060.000 ± 21.593	0.730
	M-D2	1069.000 ± 22.847	1241.330 ± 21.159	0.017
	M-D3	1069.000 ± 22.847	1320.330 ± 18.274	≤0.001
	M-D4	1069.000 ± 22.847	1426.330 ± 15.110	0.003
	D1-SD1	1060.000 ± 21.593	1078.000 ± 9.942	0.414
	D2-SD2	1241.330 ± 21.159	1083.660 ± 12.860	0.005
	D3-SD3	1320.330 ± 18.274	1271.330 ± 13.967	0.069
	D4-SD4	1426.330 ± 15.110	1390.000 ± 11.690	0.100

**Table:** (j) Expression of GPx1 in GPx1 knockdown splenic lymphocytes of pigs

GPx1 mRNA				
Time	Groups	Contents		*P*
48 h	P-M	1.032 ± 0.110	1.000 ± 0.055	0.564
	M-SE	1.000 ± 0.055	1.194 ± 0.176	0.445
	M-D1	1.000 ± 0.055	0.993 ± 0.519	0.989
	M-D2	1.000 ± 0.055	0.752 ± 0.083	0.238
	M-D3	1.000 ± 0.055	0.526 ± 0.047	0.008
	M-D4	1.000 ± 0.055	0.420 ± 0.012	0.052
	D1-SD1	0.993 ± 0.519	1.027 ± 0.537	0.225
	D2-SD2	0.752 ± 0.083	0.845 ± 0.083	≤0.001
	D3-SD3	0.526 ± 0.047	0.684 ± 0.024	0.064
	D4-SD4	0.420 ± 0.012	0.448 ± 0.098	0.724

**Table:** (k) Expression of SOD in GPx1 knockdown splenic lymphocytes of pigs

SOD mRNA				
Time	Groups	Contents		*P*
48 h	P-M	1.852 ± 0.042	1.000 ± 0.036	0.041
	M-SE	1.000 ± 0.036	1.258 ± 0.113	0.247
	M-D1	1.000 ± 0.036	0.938 ± 0.063	0.194
	M-D2	1.000 ± 0.036	0.909 ± 0.038	0.333
	M-D3	1.000 ± 0.036	0.737 ± 0.043	0.012
	M-D4	1.000 ± 0.036	0.608 ± 0.081	0.052
	D1-SD1	0.938 ± 0.063	1.156 ± 0.009	0.112
	D2-SD2	0.909 ± 0.038	1.035 ± 0.177	0.559
	D3-SD3	0.737 ± 0.043	0.902 ± 0.054	0.030
	D4-SD4	0.608 ± 0.081	0.635 ± 0.021	0.772

**Table:** (a) Expression of Bcl-2 in GPx1 knockdown splenic lymphocytes of pigs

Bcl-2 mRNA				
Time	Groups	Contents		*P*
48 h	P-M	1.067 ± 0.098	1.000 ± 0.436	0.888
	M-Se	1.000 ± 0.436	1.170 ± 0.064	0.714
	M-D1	1.000 ± 0.436	0.965 ± 0.022	0.925
	M-D2	1.000 ± 0.436	0.910 ± 0.074	0.845
	M-D3	1.000 ± 0.436	0.755 ± 0.139	0.451
	M-D4	1.000 ± 0.436	0.315 ± 0.027	0.254
	D1-SD1	0.965 ± 0.022	0.985 ± 0.010	0.554
	D2-SD2	0.910 ± 0.074	0.951 ± 0.023	0.658
	D3-SD3	0.755 ± 0.139	0.765 ± 0.090	0.814
	D4-SD4	0.315 ± 0.027	0.426 ± 0.068	0.346

**Table:** (b) Expression of Bax in GPx1 knockdown splenic lymphocytes of pigs

Bax mRNA				
Time	Groups	Contents		*P*
48 h	P-M	0.674 ± 0.012	1.000 ± 0.294	0.373
	M-Se	1.000 ± 0.294	0.856 ± 0.105	0.700
	M-D1	1.000 ± 0.294	1.166 ± 0.040	0.610
	M-D2	1.000 ± 0.294	1.022 ± 0.010	0.930
	M-D3	1.000 ± 0.294	1.795 ± 0.074	0.201
	M-D4	1.000 ± 0.294	3.762 ± 0.107	0.065
	D1-SD1	1.166 ± 0.040	0.946 ± 0.206	0.311
	D2-SD2	1.022 ± 0.010	1.033 ± 0.033	0.705
	D3-SD3	1.795 ± 0.074	1.174 ± 0.034	0.029
	D4-SD4	3.762 ± 0.107	2.502 ± 0.327	0.152

**Table:** (a) Expression of DNMT1 in GPx1 knockdown splenic lymphocytes of pigs

DNMT1 mRNA				
Time	Groups	Contents		*P*
48 h	P-M	1.389 ± 0.080	1.000 ± 0.177	0.111
	M-Se	1.000 ± 0.177	1.521 ± 0.170	0.280
	M-D1	1.000 ± 0.177	0.849 ± 0.002	0.443
	M-D2	1.000 ± 0.177	0.806 ± 0.095	0.497
	M-D3	1.000 ± 0.177	0.760 ± 0.045	0.235
	M-D4	1.000 ± 0.177	0.530 ± 0.167	0.304
	D1-SD1	0.849 ± 0.002	0.871 ± 0.033	0.493
	D2-SD2	0.806 ± 0.095	0.834 ± 0.212	0.791
	D3-SD3	0.760 ± 0.045	0.826 ± 0.083	0.246
	D4-SD4	0.530 ± 0.167	0.750 ± 0.100	0.452

**Table:** (b) Expression of DNMT3a in GPx1 knockdown splenic lymphocytes of pigs

DNMT3a mRNA				
Time	Groups	Contents		*P*
48 h	P-M	1.045 ± 0.151	1.000 ± 0.017	0.770
	M-Se	1.000 ± 0.017	1.142 ± 0.089	0.221
	M-D1	1.000 ± 0.017	0.837 ± 0.141	0.314
	M-D2	1.000 ± 0.017	0.453 ± 0.062	0.065
	M-D3	1.000 ± 0.017	0.434 ± 0.079	0.050
	M-D4	1.000 ± 0.017	0.158 ± 0.017	≤ 0.001
	D1-SD1	0.837 ± 0.141	0.901 ± 0.441	0.815
	D2-SD2	0.453 ± 0.062	0.546 ± 0.014	0.337
	D3-SD3	0.434 ± 0.079	0.469 ± 0.009	0.680
	D4-SD4	0.158 ± 0.017	0.291 ± 0.009	0.026

**Table:** (c) Expression of DNMT3b in GPx1 knockdown splenic lymphocytes of pigs

DNMT3b mRNA				
Time	Groups	Contents		*P*
48 h	P-M	1.338 ± 0.076	1.000 ± 0.036	0.053
	M-Se	1.000 ± 0.036	1.586 ± 0.166	0.152
	M-D1	1.000 ± 0.036	0.583 ± 0.049	0.013
	M-D2	1.000 ± 0.036	0.534 ± 0.041	0.074
	M-D3	1.000 ± 0.036	0.467 ± 0.024	0.010
	M-D4	1.000 ± 0.036	0.400 ± 0.007	0.021
	D1-SD1	0.583 ± 0.049	0.653 ± 0.021	0.388
	D2-SD2	0.534 ± 0.041	0.561 ± 0.004	0.554
	D3-SD3	0.467 ± 0.024	0.555 ± 0.016	0.043
	D4-SD4	0.400 ± 0.007	0.464 ± 0.033	0.174

**Table:** (d) Expression of MBD2 in GPx1 knockdown splenic lymphocytes of pigs

MBD2 mRNA				
Time	Groups	Contents		*P*
48 h	P-M	0.977 ± 0.106	1.000 ± 0.182	0.740
	M-Se	1.000 ± 0.182	0.996 ± 0.139	0.925
	M-D1	1.000 ± 0.182	0.492 ± 0.002	0.160
	M-D2	1.000 ± 0.182	2.281 ± 0.092	0.095
	M-D3	1.000 ± 0.182	2.364 ± 0.278	0.032
	M-D4	1.000 ± 0.182	4.611 ± 0.242	0.008
	D1-SD1	0.492 ± 0.002	0.390 ± 0.008	0.025
	D2-SD2	2.281 ± 0.092	2.293 ± 0.014	0.859
	D3-SD3	2.364 ± 0.278	2.297 ± 0.194	0.874
	D4-SD4	4.611 ± 0.242	3.004 ± 1.206	0.361

## Data Availability

All data used to support the findings of this study are included within the article.

## Additional Points

*Highlights*. Primary culture of porcine splenic lymphocytes in vitro. Overexpress or knockdown of GPx1 gene to explore its role in cytotoxicity of DON
